# 

**Published:** 1889-09

**Authors:** 


The Annual Announcement of the New York Polyclinic, a Clinical School for Graduates in Medicine and Surgery, shows an attendance for the session of 1888–9 of 383 physicians, making, since the opening of the pioneer post-graduate school in 1882, a total of 1883. These figures demonstrate, beyond all doubt, the popularity of the Polyclinic System of instruction. The most important feature of this year's catalogue is the *Polyclinic Hospital.* By the enlargement of their property the Faculty have established an extensive hospital which will afford at all times ample material for all clinical purposes. The Polyclinic and Hospital buildings have been completely fitted out with all the modern appliances conducive to the healthfulness and comfort of the patients and physicians in attendance.

The sessions of 1889–90 will open Monday, September 16.

### BOOKS RECEIVED.

A MANUAL OF CHEMISTRY FOR THE USE OF MEDICAL STUDENTS, by Brandreth Symonds, A. M., M. D., Assistant Physician to Roosevelt Hospital, out-patient department; attending physician, Northwestern Dispensary, New York. Philadelphia, P. Blakiston, Son & Co., 1012 Walnut street, 1889.

A LABORATORY GUIDE IN URINALYSIS AND TOXICOLOGY, by R. A. Witthaus, A. M, M. D., Professor of Chemistry and Physics in the Medical Department University of the City of New York; Professor of Chemistry and Toxicology in the Medical Department, University of Vermont; Member of the American Chemical Society, and of the Chemical Societies of Paris and Berlin, etc. Second edition. New York. William Wood & Company, 56 and 58 LaFayette Place, 1889.

QUESTIONS AND ANSWERS OF THE ESSENTIALS OF OBSTETRICS, PREPARED ESPECIALLY FOR STUDENTS OF MEDICINE, by William Easterly Ashton, M. D., Demonstrator of Clinical Obstetrics in the Jefferson Medical College; Chief of Clinic for Diseases of Women in the Jefferson Medical College Hospital; Member of the Obstetrical Society of Philadelphia, etc. With illustrations. Philadelphia, W. B. Saunders, 1888.

THE PHYSICIANS LEISURE LIBRARY. DYSPEPSIA, by Frank Woodbury, M. D., Detroit. Geo. S. Davis. 1888.

ATLAS OF VENEREAL AND SKIN DISEASES, Comprising Original Contributions and Selections from the Works of Prof. M. Kaposi, of Vienna; Dr. J. Hutchinson, of London; Prof. I. Newman, of Vienna; Profs. A. Fournier and A. Hardy; and Drs. Ricord, Cullerrier, Besnier and Vidal, of Paris; Dr. P. A. Morrow, of New York; Dr. E. L. Keyes, of New York; Dr. Fessenden W. Otis, of New York; Dr. Nevins Hyde, of Chicago; Dr. Henry G. Piffard, of New York, and others. Edited by Prince A. Morrow, A. M., M. D., Clinical Professor of Venereal Diseases, formerly Clinical Lecturer on Dermatology, in the University of the city of New York; Surgeon to Charity Hospital, etc. New York, William Wood & Company, 1888.

INEBRIETY, ITS ETIOLOGY, PATHOLOGY, TREATMENT, AND JURISPRUDENCE, by Norman Kerr, M. D., F. L. S. Fellow of the Medical Society for the study of Inebriety; Chairman British Medical Association Inebriates' Legislative Committee; Consulting Physician Dalrymple House, for the treatment of Inebriety; Corresponding Member Medico-Legal Society of New York; Corresponding Secretary American Association for the cure of Inebriates. Second Edition. London. H. K. Lewis, 136 Gower street, W. C. 1889.

